# *EGFR*突变非小细胞肺癌免疫治疗相关研究进展

**DOI:** 10.3779/j.issn.1009-3419.2019.08.11

**Published:** 2019-08-20

**Authors:** 鑫 王, 殿胜 钟

**Affiliations:** 300052 天津，天津医科大学总医院肿瘤内科 Department of Medical Oncology, Tianjin Medical University General Hospital, Tianjin 300052, China

**Keywords:** 肺肿瘤, 表皮生长因子受体, 免疫治疗, Lung neoplasms, Epidermal growth factor receptor, Immunotherapy

## Abstract

靶向治疗和免疫治疗是非小细胞肺癌（non-small cell lung cancer, NSCLC）重要治疗手段。目前表皮生长因子受体（epidermal growth factor receptor, *EGFR*）突变NSCLC临床和基础研究还处在探索阶段，需要优化免疫治疗与其他治疗方式的疗效、组合、顺序和剂量，明确EGFR突变与免疫微环境、免疫治疗疗效三者间的关系。本文就免疫治疗*EGFR*突变NSCLC临床前研究、程序性死亡受体1（programmed cell death protein ligand 1, PD-L1）表达和肿瘤突变负荷、治疗等方面最新研究进行综述。

## Abstract (en)

Targeted therapy and immunotherapy are important treatments for non-small cell lung cancer (NSCLC). At present, the clinical and basic research of epidermal growth factor receptor (*EGFR*) mutation NSCLC is still in the exploratory stage, needing to optimize the efficacy, combination, sequence and dosage of immunotherapy and other treatments, to clarify the relationship between *EGFR* mutation, immune microenvironment and the efficacy of immunotherapy. In this review, we summarized the newly updated data about immunotherapy in *EGFR* mutant NSCLC in term of pre-clinical study, programmed cell death protein ligand 1 (PD-L1) expression, tumor mutation burden and treatment.

表皮生长因子受体（epidermal growth factor receptor, *EGFR*）突变是非小细胞肺癌（non-small cell lung cancer, NSCLC）最常见驱动的基因之一，酪氨酸激酶抑制剂（tyrosine kinase inhibitors, TKIs）是*EGFR*突变NSCLC最佳治疗选择，但是耐药不可避免且机制复杂。克服EGFR-TKIs耐药手段仍有限，亟需新的更有效治疗方法。伴随免疫治疗的快速发展，作用于程序性死亡-1（programmed death 1, PD-1）受体及其配体（programmed cell death ligand 1, PD-L1）免疫检查点抑制剂成为晚期NSCLC新的标准治疗^[1-3]^。

*EGFR*突变是亚洲肺癌患者最重要分子亚型，占肺腺癌40%-50%^[4]^。患者和医生都期待免疫治疗能应用于*EGFR*突变NSCLC，但是，相关的免疫治疗基础和临床研究处在探索阶段，*EGFR*突变与免疫微环境、免疫治疗疗效三者间的关系还不明确。另外，免疫治疗的进步为*EGFR*突变肺癌提供了新研究方法。本综述介绍免疫治疗*EGFR*突变肺癌临床前研究、PD-L1表达和肿瘤突变负荷、治疗等方面数据，总结该领域进展、争议及面临的挑战。

## 1 临床前研究

早期临床前研究提示，*EGFR*突变激活PD-1通路，对PD-1/PD-L1抑制剂可能敏感。细胞系方面研究，与EGFR野生型比较，*EGFR*突变提高PD-L1蛋白表达和mRNA水平，吉非替尼可下调PD-L1蛋白表达^[5, 6]^。EGFR-TKIs增加干扰素γ分泌，减少T细胞凋亡和PD-L1表达，具有增强抗肿瘤免疫效应的作用^[6]^。体内研究进一步验证上述报道，Akbay等^[5]^利用*EGFR*突变转基因小鼠，发现PD-1抑制剂可诱导肿瘤退缩延长生存期。

临床研究数据^[2, 3, 7-10]^显示，免疫治疗*EGFR*突变NSCLC疗效差强人意，与实验室研究结果并不一致，可能与*EGFR*突变和野性型肺癌在PD-L1表达机制上存在差异有关。目前，存在两种PD-L1表达机制^[11]^，适应性免疫抵抗通过免疫浸润细胞分泌干扰素γ上调PD-L1表达，固有免疫抵抗通过癌基因信号改变诱导内生性PD-L1表达，*EGFR*突变肺癌PD-L1表达属于后者。Tang等^[12]^发现*EGFR*突变肿瘤微环境缺乏T细胞，导致PD-1/PD-L1抑制剂难以发挥疗效，*EGFR*突变NSCLC标本中极少同时出现PD-L1表达和CD8^+^肿瘤浸润淋巴细胞^[13]^。

*EGFR*突变可能在肺癌免疫逃逸和耐受上发挥核心作用，重塑肿瘤免疫微环境形成“冷肿瘤”，PD-L1低表达、肿瘤浸润淋巴细胞缺乏，低肿瘤突变负荷（tumor mutation burden, TMB）、适应性免疫抵抗缺乏等因素共同导致免疫治疗*EGFR*突变NSCLC疗效不佳^[14]^。

## 2 PD-L1表达

PD-L1是目前NSCLC免疫治疗最重要的疗效预测指标^[1]^。*EGFR*突变肺癌PD-L1蛋白表达波动范围较大，阳性率11%-73%不等^[14-16]^。*EGFR*突变肺癌PD-L1表达水平增高、还是降低，不同研究结果不一致，甚至彼此矛盾。

Azuma等^[17]^第一次报道手术切除标本*EGFR*突变与PD-L1表达间的关系，*EGFR*突变较野生型PD-L1阳性率明显增加，比值比（odds ratio, OR）为25.4。后续同样有研究显示，与EGFR野性型比较，*EGFR*突变肺癌PD-L1表达水平明显增加^[12, 18]^。但是，Ji等^[16]^发现*EGFR*突变肺癌PD-L1表达水平下降，合并分析18篇研究3, 959例患者显示，*EGFR*突变导致PD-L1表达明显下降，与EGFR野性型比较OR为0.59（95%CI: 0.39-0.92, *P* < 0.02）^[19]^，TCGA数据分析EGFR突变和PD-L1表达为反向关系^[14]^。PD-L1表达水平不一致^[5, 18]^的原因可能与人群和抗体选择、免疫组化技术、EGFR-TKIs治疗影响及不同耐药机制有关。

*EGFR*突变肺癌PD-L1蛋白表达特点有：①很少合并PD-L1强阳性表达，PD-L1表达≥50%发生率为0.5%-9.9%^[16, 20]^。②PD-L1表达与*EGFR*突变类型无关，19外显子缺失和L858R点突变在PD-L1表达上无明显差异^[15, 21]^。③不同耐药机制PD-L1表达水平不同，T790M耐药突变PD-L1表达水平下降^[22]^，T790M突变阴性和阳性比较，在PD-L表达≥1%比例上类似，但PD-L表达≥10%和≥50%比例明显增加。EGFR-TKIs耐受机制不同导致不同免疫原性，针对不同耐药机制需要个体化给予免疫治疗。④PD-L1还不能作为免疫治疗*EGFR*突变NSCLC疗效预测标志物，即使PD-L1高表达免疫治疗疗效也不理想，Keynote 001研究显示，帕博利珠单抗治疗*EGFR*突变和野生型PD-L1表达≥50% NSCLC^[23]^，客观有效率（objective response rate, ORR）分别为20%和40%，中位生存期（overall survival, OS）分别为6.5个月和15.7个月；另一项Ⅱ期研究^[10]^中帕博利珠单抗治疗8例PD-L1表达≥50%、未经TKI治疗*EGFR*突变肺癌，无1例有效。

## 3 肿瘤突变负荷

TMB是肺癌免疫治疗预测标志物^[24]^，伴随TMB增加，肿瘤免疫原性增强。与PD-L1表达水平差异较大不同，文献报道一致认为，*EGFR*突变肺癌TMB值较低^[14, 25]^，Offin等^[25]^报道中位TMB值较野生型明显下降，3.77突变/百万碱基（mutation/megabase, Mu/Mb）*vs* 6.12 Mu/Mb（*P* < 0.01）。

*EGFR*突变肺癌TMB具有以下特点：①不同*EGFR*突变亚型TMB值可能不同^[25]^，L858R点突变较19外显子缺失TMB明显增加，4.72 Mu/Mb *vs* 3.17 Mu/Mb（*P*=0.003），目前未见有关非经典*EGFR*突变TMB文献报道。②EGFR-TKIs治疗导致TMB变化^[25]^，与治疗前比较EGFR-TKIs耐药后TMB明显升高，3.42 Mu/Mb *vs* 6.56 Mu/Mb（*P*=0.008），EGFR-TKIs耐药后TMB增加为后线免疫治疗提供理论依据，但是，耐药后TMB增加是否伴有免疫原性增强有待进一步研究。③治疗前低TMB可能与继发性T790M耐药突变相关，出现和未出现T790M耐药突变基线TMB有下降趋势，3.77 Mu/Mb *vs* 4.77 Mu/Mb（*P*=0.057），低TMB患者耐药后更容易出现T790M突变。④TMB可预测EGFR-TKIs疗效，Blakely等^[26]^使用cfDNA分析突变负荷，EGFR-TKIs治疗无效患者TMB明显增加；以中位TMB为阈值^[25]^，低TMB和高TMB两组中位至EGFR-TKIs治疗中断时间分别为17个月和10个月（HR=0.56, *P*=0.006）。⑤TMB是*EGFR*突变肺癌预后因素，低TMB和高TMB亚组中位OS分别为41个月和29个月（HR=0.52,  *P*=0.03）^[25]^。

## 4 免疫检查点抗体单药治疗

### 4.1 免疫检查点抗体单药一线治疗

帕博利珠单抗单药批准用于PD-L1表达≥50%晚期NSCLC，联合化疗批准用于晚期非鳞和鳞状NSCLC^[1, 27]^。然而，免疫一线治疗的关键Ⅲ期研究中多将*EGFR*突变患者排除在外，免疫单药用于未经EGFR-TKIs治疗*EGFR*突变肺癌的数据十分有限。帕博利珠单抗一线治疗*EGFR*突变PD-L1表达≥1%晚期NSCLC单中心Ⅱ期研究^[10]^，因未达到首要研究终点ORR≥26%而提前终止入组，10例患者无1例有效，中位无进展生存期（progression free survival, PFS）为119 d。

### 4.2 免疫检查点抗体单药二线治疗

PD-1/PD-L1抑制剂，如纳武利尤单抗、帕博利珠单抗和atezolizumab，是晚期NSCLC标准二线治疗，相关Ⅲ期临床研究纳入EGFR-TKIs治疗进展*EGFR*突变患者^[2, 3, 28]^。Checkmate 057研究亚组分析，纳武利尤单抗治疗82例*EGFR*突变患者未带来PFS和OS显著改善^[2]^。在Keynote 010^[3]^和OAK^[28]^研究中观察到类似结果，尽管总体人群免疫治疗较二线多西他赛OS明显延长，但是，*EGFR*突变亚组分析，OS均无获益。回顾性数据^[13]^显示，免疫治疗*EGFR*突变或*ALK*融合与阴性患者相比，ORR明显下降，3.6% *vs* 23.3%（*P*=0.053）。

免疫单药二线治疗*EGFR*突变肺癌*meta*分析^[29]^，纳入Checkmate 057、Keynote 010和POPLAR三项研究，其中，*EGFR*突变患者186例，免疫治疗显著延长意向治疗人群和EGFR野生型患者的OS，但是，*EGFR*突变患者OS无明显延长（HR=1.05, 95%CI: 0.70-1.55, *P*=0.81）。另外一项*meta*分析^[29]^再次验证上述结果，免疫治疗EGFR野生型生存获益明显，而*EGFR*突变患者无生存获益。因此，目前美国国立综合癌症网络（National Comprehensive Cancer Network, NCCN）指南认为，*EGFR*突变NSCLC免疫治疗疗效不佳，不推荐*EGFR*突变NSCLC接受免疫治疗。

### 4.3 免疫检查点抗体单药三线及以上治疗

一代或三代EGFR-TKIs治疗失败后可供选择有效治疗手段有限，且肿瘤细胞对EGFR通路依赖性下降，为免疫治疗提供了机会。Ⅱ期ATLANTIC研究根据*EGFR*、*ALK*基因状态和PD-L1表达分为三组^[30]^，给予PD-L1抑制剂durvalumab三线及后线治疗，PD-L1表达≥25%、且*EGFR*突变亚组，ORR为12.2%；PD-L1表达 < 25%、且*EGFR*突变亚组，ORR仅为4%^[30]^。根据该研究结果，PD-L1高表达的*EGFR*突变NSCLC患者，免疫治疗可作为三线及后线治疗选择之一。

## 5 免疫检查点抗体联合治疗

### 5.1 免疫检查点抗体联合EGFR-TKIs治疗

有多项PD-1/PD-L1抑制剂联合EGFR-TKIs的Ⅰ期/Ⅱ期临床研究。在CheckMate 012研究中^[31]^，厄洛替尼联合纳武利尤单抗治疗21例*EGFR*突变NSCLC（既往接受厄洛替尼治疗20例，未经TKIs治疗1例），最常见不良反应包括皮疹、乏力、甲沟炎、腹泻，3级毒性发生率为19%，未发生4级-5级毒性，ORR为19%，24周PFS率51%。厄洛替尼联合atezolizumab治疗*EGFR*突变NSCLC Ⅰ期临床研究^[7]^，纳入患者28例，ORR 75%，中位治疗持续时间9.7个月。另一项Ⅰ期临床研究评价吉非替尼联合durvalumab用于未经EGFR-TKIs治疗NSCLC，第一组吉非替尼联合durvalumab，共10例患者；第二组吉非替尼单药治疗28 d后联合durvalumab，共10例患者。两组ORR分别为77.8%和80%^[32]^，多数患者耐受性可，两组任何级别不良反应发生率为80%和60%，但是，3级-4级谷丙转氨酶升高两组发生率分别为70%和60%，3级-4级谷草转氨酶升高发生率分别为40%和50%，谷丙转氨酶和谷草转氨酶升高可通过剂量调整和激素处理。

Ib期TATTON研究评价奥希替尼联合durvalumab方案^[33]^，T790M阳性和阴性组ORR分别为67%和21%，间质性肺炎发生率38%，3级-4级间质性肺炎15%，单药奥希替尼和durvalumab间质性肺部发生率仅为2%-3%，鉴于该联合方案显著增加肺毒性而提前终止。Oshima等^[34]^分析EGFR-TKI联合或不联合纳武利尤单抗间质性肺炎发生率，总体人群、EGFR-TKIs单药和EGFR-TKIs联合纳武利尤单抗间质性肺炎发生率分别为4.8%、4.59%和25.7%，联合纳武利尤单抗间质性肺炎风险增加4.31倍。Takakuwa等^[35]^报道1例*EGFR*突变NSCLC患者，纳武利尤单抗治疗结束后37 d给予奥希替尼治疗后出现症状性间质性肺炎，可能与其半衰期较长（纳武利尤单抗半衰期为25 d）相关。因EGFR-TKIs联合免疫治疗出现较高的不良反应，限制了它们在临床中的使用。免疫治疗与EGFR-TKIs是联合、序贯还是间插，需进一步探索。

### 5.2 免疫检查点抗体联合化疗治疗

IMpower150研究^[36]^中，EGFR/ALK阳性患者在含铂化疗加贝伐单抗基础增加atezolizumab，可带来OS显著获益，提示化疗联合免疫治疗可能是EGFR-TKIs耐药后治疗选择之一。目前正在进行Checkmate722研究（NCT02864251）纳入EGFR-TKIs治疗进展后T790M阴性晚期NSCLC，分为含铂化疗联合纳武利尤单抗、纳武利尤单抗联合ipilimumab及含铂化疗三组。类似的Keynote789研究（NCT03515837）入组EGFR-TKIs耐药后T790M阴性或一线奥希替尼进展患者，随机分为培美曲塞/卡铂组或培美曲塞/卡铂联合帕博利珠单抗，首要研究终点为PFS。

### 5.3 免疫检查点抗体联合其他药物治疗

除经典免疫检查点抑制剂抗PD-1/PD-L1单抗外，HHLA2属于新发现B7/CD28家族一员，广泛表达于肺癌细胞，值得关注的是，HHLA2在*EGFR*突变NSCLC表达水平更高，可能是*EGFR*突变患者免疫治疗新的靶向^[37]^。表达于DC细胞的吲哚胺2, 3-双加氧酶（indoleamine-2, 3-dioxygenase, IDO）在多种肿瘤中是关键的免疫耐受调节因子，抑制T细胞效应，促进免疫耐受^[38]^。Epacadostat是强效高选择性IDO1抑制剂，三期Keynote-715研究Epacadostat联合帕博利珠单抗加减化疗一线治疗NSCLC的研究正在进行。

## 6 结语

目前，*EGFR*突变NSCLC，EGFR-TKIs仍是首选治疗。由于该领域临床研究还不成熟、且*EGFR*突变NSCLC耐药机制复杂，需要优化免疫治疗与其他治疗方式的疗效、组合、顺序和剂量。此外，寻找有效疗效预测标志物是*EGFR*突变NSCLC免疫治疗一项十分重要的工作。*EGFR*突变信号通路在NSCLC免疫逃逸和耐药发挥核心，肿瘤浸润淋巴细胞缺乏和低肿瘤突变负荷等因素导致免疫治疗疗效有限，需要开展更多研究探索*EGFR*突变NSCLC肿瘤免疫微环境。
